# Interlinking journal and wiki publications through joint citation: Working examples from ZooKeys and Plazi on Species-ID

**DOI:** 10.3897/zookeys.90.1369

**Published:** 2011-04-14

**Authors:** Lyubomir Penev, Gregor Hagedorn, Daniel Mietchen, Teodor Georgiev, Pavel Stoev, Guido Sautter, Donat Agosti, Andreas Plank, Michael Balke, Lars Hendrich, Terry Erwin

**Affiliations:** 7 *Plazi, Zinggstrasse 16, Bern, Switzerland*; 1 *Institute of Biodiversity and Ecosystem Research, Sofia, Bulgaria*; 2 *Julius Kühn-Institute, Königin-Luise-Straße 19, 14195 Berlin, Germany*; 3 *Science 3.0*; 4 *Pensoft Publishers, 13a Geo Milev Str., Sofia, Bulgaria*; 5 *National Museum of Natural History, 1 Tsar Osvoboditel blvd., Sofia, Bulgaria*; 6 *IPD Böhm, Karlsruhe Institute of Technology, Germany*; 7 *Plazi, Zinggstrasse 16, Bern, Switzerland*; 8 *GeoBio Center, Ludwigs-Maximilians-Universität, München, Germany*; 9 *Zoologische Staatssammlung, Münchhausenstrasse 21, D-81247 München, Germany*; 10 *Smithsonian Institution, Washington, DC, USA*

## Abstract

Scholarly publishing and citation practices have developed largely in the absence of versioned documents. The digital age requires new practices to combine the old and the new. We describe how the original published source and a versioned wiki page based on it can be reconciled and combined into a single citation reference. We illustrate the citation mechanism by way of practical examples focusing on journal and wiki publishing of taxon treatments. Specifically, we discuss mechanisms for permanent cross-linking between the static original publication and the dynamic, versioned wiki, as well as for automated export of journal content to the wiki, to reduce the workload on authors, for combining the journal and the wiki citation and for integrating it with the attribution of wiki contributors.

## Introduction

The static character of academic publications inherited from the era of paper publishing is obviously at odds with the dynamic and interminable process of taxonomic research (Erwin and Johnson 2000, Penev et al. 2009). The Internet has enabled online publishing methods that provide straightforward ways to change published content over time. On the other side, time-stamped, non-modifiable, persistent scientific publications are one of the fundamentals of scholarly communication and publishing practices. They are important for several reasons, most importantly for a permanent publication record, citability and compliance with the biological Codes, e.g., The International Code of Botanical Nomenclature (ICBN) and The International Code of Zoological Nomenclature (ICZN). They also provide a stable publication record for any other purpose, e.g., registration of priority and academic credit, scientific trust, assessment of priority for ideas as well as for taxon descriptions, management of publication records and bibliographic indexing. Many online publication systems guarantee neither persistence nor a public traceability of version changes over time.

The idea of a combination between journal-published taxon descriptions and Internet-based updates was first proposed by Erwin and Johnson (2000) at a time when wikis were niche applications known only to few. The wiki approach was rendered popular by Wikipedia; the MediaWiki software it runs on is an environment in which users can create and edit public content. Importantly, wiki software like MediaWiki keeps a publicly accessible, time-stamped history of all changes over time, which renders it interesting from the perspective of scholarly peer review (Black 2008). Nevertheless, the worlds of scholarly publishing and wikis still exhibit little overlap, despite numerous scholars contributing to both (Page 2010) and even non-academic wikis, such as Wikipedia and Citizendium, that actively invite expert contributions (The Guardian 2011, Morris and Mietchen 2010).

In an attempt to reconcile the static character of taxon descriptions and the need to continuously update them, ZooKeys published recently the pilot article of Hendriks and Balke (2011), where the journal description of the new beetle species Neobidessodes darwiniensis was exported manually to a wiki taxon page on species-id.net on the day of publication (http://species-id.net/wiki/Neobidessodes_darwiniensis). The original journal description contains the link to the wiki page of the species, while the wiki page points to the journal publication as original source of information. Both sides will profit from such a workflow: the wiki version of the description can be further edited and expanded with new information, providing an opportunity for a potentially eternal process of improvement and data enrichment; at the same time, the journal publication validates taxa (re-)descriptions and provides a permanent publication record. The presence of the originally published source on the wiki page derived from it and the explicit requirement to cite both, will increase citation rates of taxonomic publications (see, e.g., Nielsen 2008). Low citation rates have been recognized as one of the main impediments to contemporary taxonomy (Werner 2006, Agnarsson and Kuntner 2007).

The current paper describes an improved method to (1) combine the citation of the original taxon treatment published in a journal with the versioned wiki page of it into a single bibliographic reference; (2) list the names of the contributors to the particular versioned wiki page in the same reference alongside with the date and version number; (3) automatically export taxon treatments and create wiki pages of them simultaneously with the journal publication; (4) provide a permanent cross-linking between the original publication and the respective versioned wiki pages.

The method is demonstrated by way of sample papers (Stoev and Enghoff 2011, Bantaowong et al. 2011) published in this issue. The papers serve as an example of journal publication of taxon treatments (of both newly described and redescribed taxa) that are automatically exported to wiki (species-id.net) on the day of publication. Treatments from any paper published as TaxPub XML file can easily be uploaded, too, and we illustrate that with two PhytoKeys articles published in 2010 (Kress et al. 2010, Knapp 2010). Further, we illustrate how taxon treatments from legacy literature can be exported from Plazi’s treatment repository to Species-ID. A list of the Species-ID taxon pages generated in the frame of the present project is given in Appendix 1.

## Citation of versioned documents

Currently, wiki pages like other Internet resources are usually cited as URLs, often adding a “date of accession”. Clearly, the latter has little practical meaning if the cited Internet resource does not provide a public version history. On most wiki platforms, however, each separate edit of a wiki page is versioned and time-stamped in a publicly accessible manner. This feature, along with the ease with which edits can be made, is a major factor contributing to the phenomenal success of Wikipedia and the high popularity of wiki environments in general.

**Figure 1.**
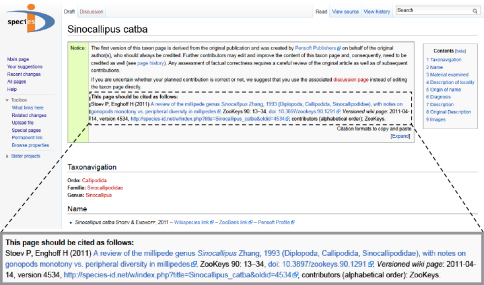
Citation template for the simultaneous journal and wiki publication of Sinocallipus catba Stoev & Enghoff, 2011 (generic link: http://species-id.net/wiki/Sinocallipus_catba, permanent link of the version depicted in the figure: http://species-id.net/w/index.php?title=Sinocallipus_catba&oldid=XXXX). The generic link always points to the most recent version of the page, while a permanent link is specific to one particular revision.

Any versioned online source, however, has the problem that two or more possible citations (and corresponding URLs) might exist, that is, the one for the most recent version and the one or more for previous versions in time. For many use cases, it is desirable to link to the most recent, presumably improved or error-corrected URL, and thus this URL is normally used when referring to an article. At arXiv and Nature Precedings, for instance, the generic identifier always points to the latest version (cf. http://arxiv.org/abs/1103.3216 and http://dx.doi.org/10.1038/npre.2009.3267), even though individual versions can technically be cited as easily (cf. http://arxiv.org/abs/1103.3216v2 and http://dx.doi.org/10.1038/npre.2009.3267.4). Both sites explain the versioning in places where few citing authors will look, which makes it difficult to establish a consistent citation pattern for different versions of the same article. Similarly, the “permanent link” available for any version of any page in a standard wiki environment (cf. Fig. 1) is not known to all users who cite wiki articles. Furthermore, attribution to the contributors to a versioned wiki page provides another barrier to a proper citation, because it requires significant wiki experience and substantial work to extract the names of the contributors from a highly edited page.

Lack of appropriate mechanisms for recognition of wiki authorship is one of the major reasons for many academics to stay away from the wiki world (cf. George 2007, Prug 2010). Clear attribution of wiki authorship is an important prerequisite for receiving credit. Thus, wiki pages intended to be cited in a scholarly manner would certainly benefit from an on-page display of a recommended citation format. In cases where the entire content has been created in an on-wiki collaboration, this is just a technical problem, for which several solutions exist (e.g., Suh et al. 2008). However, in the example of a close journal-to-wiki workflow, the first version of a page is near-identically derived from a source outside the wiki. The challenge is how to properly cite both the first, original version and the later on-wiki contributions in a single and consistent way.

Thus far, wiki elements in scholarly publishing are rare (for an overview, see Mietchen et al. 2011). In the life sciences, the two most prominent examples are RNA Biology and Scholarpedia. RNA Biology requires authors to submit the draft for a Wikipedia article along with certain types of manuscript submissions. The Wikipedia draft article is included in the peer review process and its generic wiki address (e.g., http://en.wikipedia.org/wiki/SmY, meanwhile changed to http://en.wikipedia.org/wiki/SmY_RNA) mentioned in the journal article (i.e. Jones et al. 2009), which in turn is then cited from the Wikipedia article once the journal article has been published. This way, the journal and wiki versions are interlinked yet cited independently. Scholarpedia is a review journal implemented entirely on MediaWiki. For all its accepted articles (e.g., Dawson and Lauterbur 2008), it always clearly displays the generic wiki address (i.e., http://www.scholarpedia.org/article/Magnetic_resonance_imaging), the DOI and the wiki revision number (the current one is #73087), states a recommended citation at the bottom of the page – Joan Dawson, Paul C. Lauterbur (2008) Magnetic resonance imaging. Scholarpedia, 3(7): 3381, (go to the first approved version) – and provides a prominent link on the top right to an auxiliary page containing the citation in standard bibliographic formats.

To the best of our knowledge, however, there is no established citation format that combines both an original non-wiki source and the respective wiki page within one and the same citation reference. In our understanding, such a mechanism should credit both the authors of the original publication and the contributors to the respective version of the wiki page. In addition, the reader will always be referred to a defined, numbered and time-stamped version of the wiki page that also links directly to the original published source of the wiki content.

We propose to cite wiki pages based on original scholarly publications according to the following scheme:

<Author 1, Author 2 ..... Author n> (<year>) <Title> <Journal> <IssueNo> <pages> <DOI>. *Versioned wiki page:* <YEAR-MM-DD>, version <sequential number of revision>, http://species-id.net/wiki/index.php?title=Genus_species&oldid=<sequential number of revision>, contributors (alphabetical order): Contributor 1, Contributor 2 ..... Contributor N.

The citation style is automatically added to the top of each page on Species-ID by means of a template, as illustrated in Fig. 1.

The features of the proposed citation mechanism are outlined below:

1. The citation of both the original publication and the wiki page is embedded in the automated export to the wiki.

2. The names of contributors are automatically extracted from the page history and displayed on the versioned wiki page that a user is viewing. This list includes only users who contributed to the currently displayed version of the wiki page, as well as those who have contributed to all earlier versions.

3. The version number is unique within the respective wiki (it is a consecutive revision number for all revisions of all pages on the same wiki).

4. The date field lists the date of creation of the respective version (the date when the page has been accessed is available separately).

5. The “page history” link allows a quick overview of all revisions made on the paper, usually listing also the respective time stamp and author, along with a short summary of the revision.

6. Furthermore, as a service to the reader, ready-to-copy citations in BibTeX, RIS (Endnote) and wiki (Wikipedia, Citizendium) formats are provided, and supplementary formats can easily be added in.

Furthermore, this way of citation could be applied not only to recent publications but also to historical literature from where original taxa descriptions could be extracted, marked up and exported to the wiki for further updates, as shown in the examples from Plazi (Figs 3, 4).

## Automated export to a wiki

Pensoft routinely publishes XML versions of the journal papers based on the TaxPub extension of the NLM DTD (National Library of Medicine’s Document Type Definitions format) (Catapano 2010). The XML-based markup process is embedded in the editorial practice of Pensoft (see Penev et al. 2010) and permits the tagged content to be fragmented in a way that separate parts of an article can be exported, alongside with the relevant citation metadata, onto various platforms, for example Encyclopedia of Life (EOL), the Global Biodiversity Information Facility (GBIF), Plazi and Species-ID. The XML-tagged text also provides the basis for several kinds of semantic enhancements to the published text to facilitate reading, internal cross-linking and external links (Shotton 2009, Penev et al. 2010).

To automate the export to a wiki environment, in this case Species-ID, we developed a tool, named Pensoft Wiki Convertor (PWC), which converts the XML versions of the papers into MediaWiki-based wiki pages. The PWC also converts the internal structure of taxon treatments (e.g., Type Location, Description, Distribution, Etymology and others) into sections of the wiki page. In addition, it converts reference lists and identification keys, exports taxon images and places them on the wiki page. At the same time, the PWC uses several wiki templates to facilitate the display of recommended citations, taxonomic classifications and other content elements in a way that is consistent across the site and easily editable as well. Several of the hyperlinks present on the ZooKeys article – e.g., ZooBank LSIDs, georeferenced coordinates and others – are also transferred to and displayed on the wiki page.

**Figure 2.**
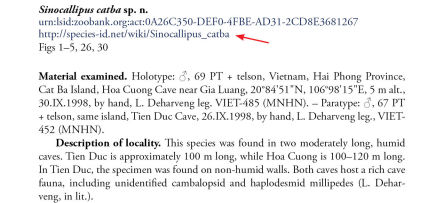
The original description of Sinocallipus catba Stoev & Enghoff, 2011 displaying the generic URL of the wiki page (http://species-id.net/wiki/Sinocallipus_catba) right below the ZooBank LSID (see arrow).

Conversely, the hyperlinked URL of the generic wiki page for each taxon treatment is published in the original journal publication, right next to the taxon treatment name (Fig. 2).

Additional wiki templates embedded by the PWC provide various links of the taxon name to external biodiversity resources (e.g., GBIF, EOL, NCBI, PubMed, Biodiversity Heritage Library (BHL), ZooBank, the International Plant Name Index (IPNI), Index Fungorum, Tropicos, PLANTS, Wikispecies, Wikipedia and others) harvested “on the fly” through the Pensoft Taxon Profile (PTP) tool.

Once the XML file of a paper is converted into MediaWiki markup, the PWC uses wiki bots to automatically create separate wiki pages for each taxon treatment and to upload the respective content there.

## The Species-ID wiki

Species-ID (http://species-id.net) is dedicated to collecting and integrating open taxon descriptions and identification tools for different taxa. The audience addressed are scientists and naturalists, both amateurs (Pearson et al. 2011) and professionals. The huge task of providing adequate documentation of the world’s biota requires a collaborative approach. The project is presently in an initial phase. Nonetheless, it already provides a sufficient infrastructure and sample pages to illustrate its mission.

A successful wiki platform can exist and develop only if there is an active community contributing to it. Several layers of contributions are welcome to Species-ID:

● Descriptions and identification tools (species treatments, dichotomous, polytomous, multi-access keys, etc.).

● Checking, editing and updating of existing wiki pages.

● Enhancing the access and usability through restructuring, categorizing, semantic wiki information or tools, wiki templates or adding new software extensions.

Species-ID publishes materials under an open content policy that is compatible with other open content projects such as Wikipedia or Open educational resources (OER). It does not resort to the “non-commercial” clause (which is highly problematic and not an open source license, see Hagedorn 2011). Unlike Wikipedia, which is dedicated to summarizing information previously published elsewhere, original and authored information may be published on Species-ID. Despite this policy, for major revisions and all nomenclatural acts, a publication in a journal is recommended. Many smaller contributions, insights, and other modifications, however, may not warrant a journal publication and are welcome on Species-ID directly. The submission of raw data files for interactive identification keys (e. g. in DELTA, Xper, SDD, or other formats) is especially encouraged to provide options for a future re-use of data.

The ability to publish independent research implies the possibility of conflict. Contributions on Species-ID may therefore either be shared, normal pages which are dedicated to neutrality, or authored pages (having the authors’ names in the title), which may support the views of the respective authors in polite discourse.

## Export of taxon treatments from legacy literature

Mark up and digitization of historical literature is a widely discussed problem in taxonomy (Agosti 2006, Agosti and Egloff 2009). The main goal of such initiatives is to bring taxonomic information published on paper to a new life and to re-use it through scanning, tagging and indexing technologies, so that it becomes fully searchable and retrievable by machines.

There are two working groups that already provide methods and tools for mark up of taxonomy literature: Plazi (based on the taxonX XML schema, see http://sourceforge.net/projects/taxonx and Sautter et al. 2007) and Inotaxa (based on the taXMLit schema, see Weitzman and Lyal 2007). Plazi has an organised taxon treatment repository and maintains it at www.plazi.org, alongside of associated services for dissemination of the published treatments.

**Figure 3.**
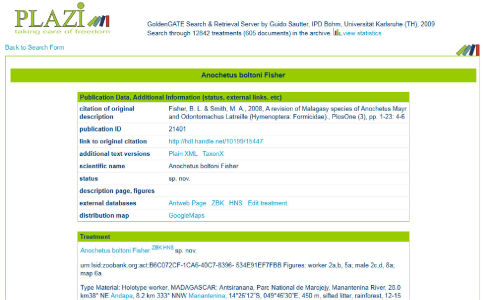
Treatment of Anochetus boltoni Fisher extracted through XML markup from the original paper of Fisher and Smith (2008) and deposited at the Plazi Treatment Repository (www.plazi.org).

**Figure 4.**
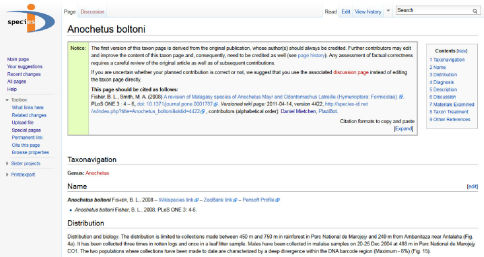
Wiki page of Anochetus boltoni Fisher (http://species-id.net/wiki/Anochetus_boltoni) exported from the Plazi Treatment Repository to Species-ID.

Using an exporting tool similar to the PWC, Plazi provided several sample treatments on species-id.net (Appendix 1). In this way, treatments from the historical literature that are available in the Plazi repository (Fig. 3) could be opened up for updating and editing, bearing at the same time the original citation details on the wiki page (Fig. 4).

## Conclusions

The present paper describes a workflow that will positively affect the exposition and dissemination of taxonomic information through:

1. Reconciliation of “static” and “dynamic” versions of a published treatment on a dedicated wiki page hosted on species-id.net;

2. Increasing exposition, discovery and linking of published information in an additional and important Internet environment, MediaWiki;

3. Combination of citation for both the original source and derivative wiki pages into a single reference record;

4. Provision of attribution to both the author(s) of the original publication and the contributor(s) to the versioned wiki page;

5. Possibility to update and edit treatments on wiki, which should provide a continuous accumulation of new knowledge;

6. Newly published and legacy treatments will be brought together to a common format for further editing, updates and possibly mashups;

7. Automating the upload to wiki will facilitate a large scale accumulation of treatments on species-id.net.

8. Wiki treatments can easily be transferred to other wikis, e.g., to Wikipedia.

We are convinced that the proposed approach will also positively influence the process of opening up data and knowledge in biodiversity science.

## Appendix 1.

List of the Species-ID pages generated automatically from the original source for the purposes of the pilot project described here.

Source: Stoev and Enghoff 2011

http://species-id.net/wiki/Sinocallipus

http://species-id.net/wiki/Sinocallipus_catba

http://species-id.net/wiki/Sinocallipus_deharvengi

http://species-id.net/wiki/Sinocallipus_jaegeri

http://species-id.net/wiki/Sinocallipus_simplipodicus

http://species-id.net/wiki/Sinocallipus_steineri

http://species-id.net/wiki/Sinocallipus_thai

Source: Bantaowong et al. 2011

http://species-id.net/wiki/Amynthas_phatubensis

http://species-id.net/wiki/Amynthas_tontong

http://species-id.net/wiki/Amynthas_borealis

http://species-id.net/wiki/Amynthas_srinan

Source: Kress et al. 2010

http://species-id.net/wiki/Larsenianthus

http://species-id.net/wiki/Larsenianthus_wardianus

http://species-id.net/wiki/Larsenianthus_careyanus

http://species-id.net/wiki/Larsenianthus_assamensis

http://species-id.net/wiki/Larsenianthus_arunachalensis

Source: Knapp 2010

http://species-id.net/wiki/Solanum_kulliwaita

http://species-id.net/wiki/Solanum_dillonii

http://species-id.net/wiki/Solanum_oxapampense

http://species-id.net/wiki/Solanum_verecundum

Source: Plazi Treatment Repository

http://species-id.net/wiki/Nixonia_masneri

http://species-id.net/wiki/Anochetus_boltoni

http://species-id.net/wiki/Formica_herculeana

http://species-id.net/wiki/Camponotus_imitator

http://species-id.net/wiki/Chromis_abyssus

http://species-id.net/wiki/Phrynoponera_pulchella
